# Responding to Suicide Clusters in the Community: What Do Existing Suicide Cluster Response Frameworks Recommend and How Are They Implemented?

**DOI:** 10.3390/ijerph19084444

**Published:** 2022-04-07

**Authors:** Nicole T. M. Hill, Jo Robinson

**Affiliations:** 1 Telethon Kids Institute, Nedlands, WA 6009, Australia; 2 Centre for Child Health Research, The University of Western Australia, Nedlands, WA 6009, Australia; 3 Orygen, Parkville, VIC 3052, Australia; jo.robinson@orygen.org.au; 4 Centre for Youth Mental Health, The University of Melbourne, Parville, VIC 3052, Australia

**Keywords:** suicide clusters, suicide prevention, cluster prevention, community response

## Abstract

Suicide clusters involve an excessive number of suicides, suicide attempts, or both, that occur close in space or time or involve social links between cluster members. Although suicide clusters are rare, evidence documenting the implementation of suicide cluster response activities in communities is required yet remains limited. In this study, we identified the core components of existing suicide cluster response frameworks through a search of the grey literature and conducted an international survey to assess the implementation of the core components by stakeholders with experience responding to a suicide cluster. The following six core components were identified from five cluster response frameworks and were incorporated into a survey assessing stakeholders’ experiences of responding to a suicide cluster: (1) Preparing for a suicide cluster; (2) Routine monitoring of suicide, suicide attempts, and cluster detection; (3) Coordination with the media and monitoring social media; (4) Identifying and supporting individuals at risk; (5) Promoting help-seeking and building community resilience; and (6) Long-term follow-up and evaluation. Twenty-six stakeholders completed the online survey. Many of the core components were implemented by stakeholders. However, gaps in practice were reported in terms of cluster surveillance, monitoring of referral uptake among bereaved individuals, and long-term evaluation. Barriers to implementation included the perceived availability and suitability of mental health services, and availability of long-term funding. Strategic policy and planning that addresses the practice-based experiences of communities has the potential to facilitate a more coordinated and timely response to suicide clusters.

## 1. Introduction

A suicide cluster is defined as an excessive number of suicides, suicide attempts, or both, which occur in close temporal and geographic proximity or among individuals who share social links (e.g., as friends or acquaintances) [1,2]. The prevalence of suicide clusters is sensitive to the methods used to detect them [2]; however, it is estimated that they make up between 1 and 13% of suicides [1,3]. The mechanisms underlying suicide clusters are not well understood [3]; however, exposure to suicide both directly (e.g., via the loss of a loved one) or indirectly (e.g., via media and social media) is often the source of significant concern among members of the community [1]. Although rare, the trauma associated with suicide and the impact of multiple suicide deaths in a community can lead to prolonged grief and widespread fear and anxiety, particularly with regards to the prospect of further suicides [4,5]. Together, these factors have been associated with increased incidence of self-harm and suicide within communities, including the maintenance of suicide clusters [1].

To facilitate a coordinated response to suicide clusters, frameworks for the detection of, response to, and prevention of suicide clusters have been developed. Although it is not known precisely how many countries have developed frameworks for responding to suicide clusters, the prevention of suicide clusters is identified as an important goal in postvention guidelines published by the World Health Organization [6]. Further, cluster response frameworks have been developed in countries including Australia, the UK, and the US [7,8,9,10] by postvention service providers and academics with a background in suicide cluster research. The purpose of these frameworks is to provide support to those immediately bereaved by suicide, as well as to facilitate the coordination of community-wide suicide prevention interventions.

Although multiple frameworks for responding to suicide clusters exist, it is unclear to what extent these frameworks overlap, and what is considered best practice when responding to a suicide cluster. Robinson and colleagues [11] described five main characteristics of national cluster response frameworks for the general population. Common features shared by these resources include: (1) Preparing for a suicide cluster; (2) Identifying that a cluster is developing via routine monitoring of suicide deaths and, where applicable, media reporting of a suicide death; (3) Responding to the cluster and providing the support or assistance required; (4) Stepping down the response; and (5) Longer-term follow-up and evaluation. However, the identification of these core components was based on what was considered by the authors to be best practice.

Although limited, there is some evidence that existing cluster response frameworks are effective in the management of suicide clusters. For example, Lai and colleagues [12] evaluated a community response to a suicide cluster in Hong Kong using a cluster response framework developed by the Centers for Disease Control and Prevention [9]. In this study, the authors reported a significant reduction in suicides following the implementation of the cluster response framework compared to the five years prior to implementation. Furthermore, qualitative interviews with stakeholders revealed that implementation was associated with increased mental health awareness, the mobilisation of community resources, and improved collaboration between community stakeholders [12]. In contrast, stigma and indifference towards marginalised members of the community were identified as a key barrier among some stakeholders tasked with responding to the suicide cluster [12].

To date, three community evaluations have examined the effectiveness of cluster response activities during a suicide cluster [13,14,15]. In general, the stakeholders responsible for responding to suicide clusters involve professionals from community postvention services, community mental health services, local police, school officials, and other organisations in the community that have contact with people at risk of suicide [7,8,9,10,13,14,15]. Common barriers that were identified by stakeholders included the absence of protocols involving the sharing of sensitive information about the deceased (i.e., history of mental health and contact with clinical services) between stakeholders from different organisations in the community; coordination with the local media and the role of social media; availability of resources to respond to the increased demand for mental health referrals in the community; implementation of sustainable suicide prevention activities in the community; provision of clinical supervision and support for stakeholders with vicarious trauma. Whilst these reports provide important insight into some of the facilitators and barriers associated with implementing a cluster response in the community, evidence within the peer-reviewed literature remains limited.

The present study sought to address these gaps in evidence in by (1) systematically identifying the core components of existing suicide cluster response frameworks and (2) conducting an international survey with stakeholders who had direct experience of responding to a suspected or actual suicide cluster. In doing so, this study sought to strengthen the evidence base with regards to what is considered best practice. The findings from this study summarise key actions and strategies for responding to suicide and provide key insights into the challenges associated with implementing these actions. The findings reported in this study have important implications for stakeholders and decision makers who are tasked with planning for and preventing suicide clusters in their community.

## 2. Materials and Methods

This study involved two parts. Part 1 involved a search of the grey literature to identify existing suicide cluster response frameworks to identify their core components (overlapping themes) and inform the key areas to be examined in a survey assessing stakeholders’ experiences with responding to a suicide cluster. The grey literature was sought due to the absence of frameworks for responding to suicide clusters published in the peer-reviewed literature [1].

Part 2 involved a cross-sectional survey delivered to an international sample of stakeholders with experience of responding to a suicide cluster. Approval from the University of Melbourne Human Research Ethics Committee was obtained for the survey (HREC 1954027).

### 2.1. Part 1: The Core Components of Cluster Response Frameworks

A search of the grey literature (using Google search engines in Australia, NZ, UK, USA, and Canada) was undertaken using the following search strategy: (“suicide*” OR “self-harm*” OR “self-inj*”) AND (“cluster” OR “contagion” OR “imitation” OR “suggestion”) AND (“prevent*” OR “community response” OR “coordinated response” OR “guideline*” OR “framework” OR “strategy” OR “respon*” OR “plan” OR “protocol”). The search engine Google was chosen as it was considered a common and accessible platform that stakeholders in the general population might seek when searching for information on suicide clusters. Links from the first 10 pages of each search (representing 100 results) were reviewed, using information from the title and underlying text. This number of pages was chosen to capture websites that were ranked as most relevant, while being a feasible amount to screen.

Eligible suicide cluster response frameworks were those which provided recommendations or actions for the detection of, response to, and/or prevention of (1) a suspected or actual suicide cluster or (2) an episode of suicide or self-harm contagion following exposure to two or more suicide deaths in the community. Cluster response frameworks specific to school, university, employment, or hospital settings were considered out of scope if they did not include strategies relevant to the general population. If a cluster response framework was superseded, only the most recent version was included. One reviewer (NTMH) conducted initial eligibility screening based on titles and abstracts in consultation with the senior authors (JR).

The core components of existing cluster response frameworks were identified by thorough thematic content analysis of the overlapping themes and actions included in the framework [16]. For the purpose of this study, a core component was defined as any theme that was present in at least 50% of the cluster response frameworks that were identified by the grey literature search. For each framework, information on the location, definition of a suicide cluster, and community settings were recorded. The core components were incorporated into a survey assessing stakeholders’ experiences of responding to a suicide cluster. Further details of the survey are described in Section 2.2.

### 2.2. Part 2: Stakeholder Survey

A custom survey was designed to examine the effectiveness of implementing the six core components identified in Part 1, followed by the barriers to implementation that were associated with each component. The resulting survey included a combination of closed (forced-choice) questions and open-ended questions asking participants whether each of the six core components were implemented (‘Yes’, ‘No’, ‘I don’t know’). Participants’ responses to the forced-choice questions were collapsed into binary outcomes representing the implementation or nonimplementation of the core component. Barriers to implementation were assessed using a list extracted from previous evaluations of cluster response activities. The list of previously encountered barriers included: (1) the absence of leadership to coordinate the relevant activities; (2) the activity was not seen as a priority; (3) there were limited financial or staffing resources; (4) lack of knowledge or experience with suicide clusters; (5) concerns about privacy or sharing information between organisations; (6) there was not enough time. Participants were asked to select any of the barriers that were relevant to their experience. A free text box was available at the end of each list, prompting participants to identify any additional barriers that they had experienced. Details of the core components are presented in the Results.

#### 2.2.1. Participants and Procedure

Data were collected via an online survey conducted between 25 July 2019 and 1 December 2019. Authors of peer-reviewed studies and authors of cluster response frameworks were identified via a custom search of the peer-reviewed and grey literature on suicide and self-harm clusters and were supplemented by the authors’ knowledge and expertise in the field. Furthermore, study information was emailed to members of the International Association for Suicide Prevention suicide clusters and contagion Special Interest Group. Lastly, study information was promoted on the social media platform Twitter by authors NH and JR. Eligible participants were individuals from any country who were 18 years and older and had been involved in (1) the development of a community suicide cluster response strategy and/or (2) a coordinated community activity involving the detection and surveillance of, response to, and/or management of a suicide cluster (e.g., the person was involved in the detection and surveillance of, response to, or management of a suicide cluster, or participated in a working group, committee, or other coordinated body that was developed or convened for the purpose of responding to a suspected or actual suicide cluster in the community). All participants had access to the survey for a 30 day period. Reminders were sent to eligible participants at two and three weeks following consent.

#### 2.2.2. Statistical Analysis

Descriptive statistics for each of the core components were analysed in R version 3.4.2 and barriers associated with implementation were reported from the open-ended questions in free-text form and were analysed using thematic content analysis [17,18]. Responses to the free-text part of the survey were grouped according to divergent and convergent themes and, where possible, frequencies of convergent themes were reported.

## 3. Results

### 3.1. Part 1: The Core Components of Cluster Response Frameworks

#### 3.1.1. Characteristics of Included Articles

The initial search of the grey literature identified 141 potentially eligible records, of which six met the inclusion criteria. A total of five cluster response frameworks were identified from Australia [10], Ireland [19], New Zealand [7], England [8], and the US [9]. One framework published by Public Health England [8] was excluded as it had been superseded by a revised version [8]. All cluster response frameworks were identified in the grey literature search [7,8,9,10,19]; however, one was also published and available in the peer-reviewed literature [9]. The included cluster response frameworks were developed in consultation with clinical and postvention service providers and clinicians with experience in responding to suicide and suicide clusters [7,8], and with academics who have expertise in suicide clusters [7,8,10]. Two frameworks did not report how they were developed [9,19].

All cluster response frameworks defined suicide clusters as multiple suicide deaths occurring closer in space and time than expected; two frameworks included both suicide and suicide attempts or self-harm events [7,8], and two frameworks described potential connections between people who died by suicide (e.g., suicide among friends or family members) in the definition of a suicide cluster [8,9]. One framework included responses to suicide clusters and murder suicides [19]. All cluster response frameworks targeted individuals in the general population and two provided culturally specific examples for implementing cluster response strategies among Aboriginal and Torres Strait Island [10] and Māori people [7]. All frameworks provided examples, resources, and recommendations for responding to suicide clusters in school settings [7,8,9,10,19].

#### 3.1.2. Core Components of Cluster Response Frameworks

Table 1 describes the six core components that were identified across cluster response frameworks, which were as follows: (1) Preparing for a suicide cluster (5/5 frameworks) [7,8,9,10,19]; (2) Routine monitoring of suicide, suicide attempts, and cluster detection (3/5 frameworks) [7,8,10]; (3) Coordination with the media and monitoring social media (5/5 frameworks) [7,8,9,10,19]; (4) Identifying and supporting individuals at risk (5/5 frameworks) [7,8,9,10,19]; (5) Promoting help-seeking and building community resilience (5/5 frameworks) [7,8,9,10,19]; and (6) Long-term follow-up and evaluation (4/5 frameworks) [7,8,10,19]. Further information on the core components included in each framework is available in Table S1 in the Supplemental Information.

### 3.2. Part 2: Results of the Stakeholder Survey

Of the 141 individuals that underwent screening, 104 did not meet the inclusion criteria. The majority of people who were excluded had knowledge of a suicide cluster in their community but had not had any experience in either planning a response or actively responding to a suicide cluster. Thirty-seven individuals were eligible to participate in the survey. Of these, four (10.8%) did not give consent to participate and an additional five (21.2%) individuals did not complete the survey. Two out of the remaining 28 eligible participants had experience of developing a cluster response plan but did not have experience responding to an actual or perceived suicide cluster. They were subsequently excluded from the analysis as their experience was qualitatively and quantitatively different to those who had been involved in the response to a local suicide cluster. The final sample comprised 26 (70.3%) completed responses from individuals who had experience of responding to an actual or perceived suicide cluster in the community.

Of the 26 study participants who completed the survey, the majority were from Australia (73%), followed by Europe (12%), New Zealand (8%), and the United States (8%). Just under half of participants had experience as a coordinator, manager or chair of a working group overseeing the response to a local suicide cluster (44%), a further 32% were involved in a working group as a stakeholder or service provider, 16% were involved as an academic or epidemiological investigator, 16% provided clinical support or were involved as a social worker, and 8% were postvention service providers. Five (19%) participants had multiple roles (e.g., both a coordinator and clinician).

#### 3.2.1. Preparing for a Suicide Cluster

Overall, 92% of participants reported having access to a pre-existing suicide cluster response framework at the time the suicide cluster emerged (Table 2). Sixty-nine percent of participants reported access to a lead agency who was responsible for developing a cluster response framework and coordinating cluster response activities in the community. Barriers to assigning a lead agency included lack of consensus with regards to who the lead agency should be (40%), followed by limited knowledge and experience in responding to suicide clusters (15%), limited resources and human capital (15%), and lack of understanding and buy-in at an organisational/agency level (13%). One participant noted in the free text that the community preferred to share the responsibility as a “whole of community issue” rather than assigning a lead agency. Other barriers identified in the free text included a lack of confidence in suicide prevention and privacy concerns (i.e., the sharing of sensitive information such as the individual’s name and peer network).

#### 3.2.2. Routine Monitoring and Cluster Detection

Overall, 35% of participants were notified of the suicide cluster via official reporting pathways established through police, coroner, or hospital admissions data, or the local health department. Instead, most participants were alerted to the potential suicide cluster via word of mouth, for example via stakeholders in clinical services, schools, and the media. Barriers associated with the monitoring of suicide and suicidal behaviours in the community were mostly attributed (65%) to the absence of the systematic collection of suicide or self-harm data in the community and the time required to access these from official sources, such as the health department or state coroner.

Participants were asked to identify sources of information that would have assisted with the planning and monitoring of suicides in the community but that were not available at the time of the suspected or actual suicide cluster. Of the 15 participants who responded, 53% wanted more timely access to suicide and self-harm data from services such as police, emergency departments, and primary care centres, and 30% wanted access to information on the social links (e.g., friendship networks) of those involved in the suicide cluster. The remaining participants wanted more information on how to identify connections between individuals via social media (7%) and wanted access to a single point of contact to obtain more timely access to suicide and self-harm data (e.g., direct correspondence with a representative from the police or local emergency department).

#### 3.2.3. Coordination with the Media and Monitoring Social Media

Most participants reported the implementation of a media strategy for the safe reporting of suicide during the suicide cluster (80%). However, 42% of participants indicated that there was no specific person or official role (e.g., an information coordinator) responsible for overseeing media-related activities. Of the 21 participants who implemented a media strategy, all of them accessed and promoted existing guidelines for the safe reporting of suicide in the media. Most participants reported that their local media adhered to existing media reporting guidelines; however, 11% of participants noted that despite local efforts, they had no control over media reports that were released by national media bodies and that these did not align with existing guidelines for the reporting of suicide in the media. Just under half of the participants reported coordinated efforts to provide training and resources to media professionals in the community on the safe reporting of suicide (47%).

Overall, 58% reported monitoring of social media memorial pages devoted to the deceased to identify potentially harmful conversations about suicide and identify individuals that were potentially at risk. Fifty-seven per cent reported signposting help-seeking information on social media platforms during the suicide cluster and two individuals (15%) disseminated help-seeking information on social media platforms in the form of paid advertisements that targeted the postcodes and demographic profiles (e.g., age and sex) of individuals that were perceived to be at risk. The main barriers reported by participants included a lack of leadership and uncertainty regarding who should monitor social media activities (35%), followed by limited knowledge and resources on how to monitor social media activities during a suicide cluster (35%). Other participants indicated that social media was not viewed as relevant to the suicide cluster at the time (17%).

#### 3.2.4. Identifying and Supporting Individuals at Risk

Participants reported that the relatives and close friends of the deceased were frequently contacted and provided bereavement support (92%). More than half of (69%) participants knew of specific procedures in place to identify and screen high-risk individuals and groups within the community (e.g., students in schools, universities). These procedures typically involved consulting with stakeholders (e.g., teachers to identify individuals that were considered to be experiencing significant distress as a result of the suicide or were otherwise known to existing mental health or other community services). A minority of participants (20%) reported using a Circles of Vulnerability model [20] in consultation with stakeholders and bereaved individuals to identify others within the community who might need postvention assistance.

Overall, 83% of participants reported knowledge of referral pathways to mental health services for those experiencing distress. However, only 23% had a system or procedure in place to monitor whether individuals who were experiencing distress made contact or engaged with mental health services as a result of the referrals that were provided.

Barriers associated with supporting individuals in distress included concerns about the limited capacity of service providers to meet the increase in consumer demand, combined with the high rates of false positives associated with suicide risk assessment. Others identified limited availability of postvention services in the community and did not think that referral to mental health services was an appropriate solution.

#### 3.2.5. Promoting Help-Seeking and Building Community Resilience

Over half (58%) of participants implemented strategies to promote help-seeking during memorial activities and at memorial sites within the community (e.g., help-seeking signage at shrines and public memorial sites and help-seeking information in funeral booklets). No specific barriers were identified; however, one participant noted that signage and barriers at suicide hotspots are required, but the messaging should be evidence-based.

Gatekeeper training was frequently provided to members of the community (80%). Seventy-seven percent of participants engaged gatekeepers (e.g., teachers, sports coaches, tribal elders) to help identify individuals who might be at risk of suicide.

Overall, 42% of participants were provided an opportunity to debrief and were encouraged to take care of their own wellbeing in response to the suicide cluster.

#### 3.2.6. Long-Term Follow-Up and Evaluation

Long-term follow-up of individuals bereaved by suicide (e.g., on significant dates such as 12 month anniversaries and birthdays) was reported by 67% of participants. Long-term evaluation of cluster response activities in the community was reported by 47% of participants. Barriers to the long-term evaluation of cluster response activities included waning commitment among stakeholders because of exhaustion and burn-out, limited resources, and limited access to long-term funding. One person noted that the absence of a readily available template to facilitate the evaluation of a local response to a suicide cluster was a significant barrier to implementation.

## 4. Discussion

To our knowledge, this is the first study to benchmark the experiences of stakeholders against the core components included in existing cluster response frameworks identified in the grey literature. The study showed that although many of the core components of existing cluster response frameworks were implemented, some gaps in best practice were identified. In particular, two-thirds of stakeholders did not have access to a data surveillance system to facilitate the timely identification of suicide and suicidal behaviours and three-quarters of stakeholders did not report a procedure for monitoring the uptake of referrals among bereaved individuals within the community. Lastly, the long-term evaluation of cluster response activities was reported among fewer than 50% of stakeholders, highlighting a clear priority for future cluster response strategies.

The barriers experienced by stakeholders who have responded to a suicide cluster provide insight into key areas for public health and policy improvement. For example, although existing frameworks recommend the monitoring and surveillance of suicide and self-harm data as part of routine cluster preparedness, detection, and management, most stakeholders became aware of the potential suicide cluster via word of mouth among stakeholders and concerned members of the community. On one hand, access to real-time data on suicide is challenging, since factors such as the police investigation and coronial process means that official confirmation of cause of death, including suicide intent, can take up to two years to determine [21,22]. Other factors such as the limited sensitivity of ICD-10 codes to the full range of self-harm behaviours pose significant challenges for the use of emergency department data in suicide and self-harm cluster surveillance [23].

To date, several suicide and self-harm surveillance systems have been developed, including the National Self-harm Registry Ireland [24], the Thames Valley Real Time Suicide Surveillance System [25], and the Victorian self-harm monitoring system [23]. However, the effectiveness of these surveillance systems in responding to suicide clusters at a community level has, to the best of our knowledge, not been evaluated. Given that suicide prevention efforts are often resource intensive, investment in coordinated suicide and self-harm surveillance systems has the potential to improve evidence-based decision making by directing resources to where they are most needed and has the potential to reduce the misuse of resources (e.g., following unverified or anecdotal reports of suicide clusters). Implementation of suicide and self-harm surveillance within the community also facilitates the long-term evaluation of activities that are implemented within the community in response to a suicide cluster.

In the present study, more than 80% of participants had implemented strategies for identifying and referring individuals to mental health or postvention support in the community. Nonetheless, few stakeholders were aware of there being a system in place to monitor whether individuals who were provided referrals then went on to engage in postvention support. Previous studies have shown that factors such as psychological distress, suicide-related stigma, and the availability of support services may significantly impact help-seeking behaviour among those bereaved by suicide [26,27]. Further, the results of the survey revealed that the provision and uptake of referrals may be equally impacted by the perceived relevance of mental health services in providing postvention support to individuals bereaved by suicide. Another study by Ross et al. [27] investigated the support needs of individuals bereaved by suicide and found that in addition to psychological and social support, assistance with practical support (e.g., forensic cleaning and funeral, legal, and financial services) were equally important. Taken together, the current study suggests that stakeholders tasked with responding to a suicide cluster ought to consider the psychological, social, and practical needs of individuals bereaved by suicide and implement a strategy to monitor the uptake of services among those bereaved.

In addition to the barriers associated with implementing the core components of suicide cluster monitoring and response, the results of the present study highlight some of the innovative interventions that communities have developed in response to suicide clusters. For example, some stakeholders described using advertising features on social media platforms (i.e., Facebook) to reach demographically relevant subgroups within the population and target them with help-seeking messages (e.g., the national Lifeline crisis support number). The use of community-led targeted advertisements can be used to refer individuals to community specific postvention services and could be adapted to disseminate evidence-based resources such as guidelines for the safe communication about suicide online [28] and related resources for the online management of suicide clusters [29].

The present study has several limitations. First, the identification of suicide cluster response frameworks was conducted by one reviewer in consultation with the senior author, but was not double-screened by a second reviewer. Further, since the core components were identified via a single search of the grey literature using the Google search engine, it is possible that some cluster response frameworks were omitted and were therefore not included in the stakeholder survey.

In terms of the stakeholder survey the number of participants who completed the survey was small. In particular, due to the small sample size, the results of the survey should be interpreted with caution as a 1 unit change represents approximately 4% of survey response rates. Nonetheless, given that suicide clusters are a particularly rare event, accounting for 1.3 to 13% of suicides [1], recruitment of participants with experience of responding to suicide clusters can be challenging. Additionally, the selection bias resulting from online surveys makes it difficult to determine the representativeness of the sample. It is therefore likely that participants were from communities with adequate resources to facilitate a response to a suicide cluster. Finally, most of the sample was Australian, and all assessments were based on self-reporting.

## 5. Conclusions

Although rare, suicide clusters are often the source of significant concern in communities when they occur. Although frameworks for the management and prevention of suicide clusters exist, there remain limited evaluations of cluster response activities that can be used to inform future prevention efforts. The current study provides insight into some of the implementation barriers experienced by stakeholders. Addressing these barriers proactively through strategic policy and planning has the potential to facilitate a more coordinated and timely response to suicide clusters and the translation of evidence into practice.

## Figures and Tables

**Table 1:** Core components of cluster response frameworks.

Cluster Response Component	Example
Preparing for a suicide cluster	Assign a lead agency to oversee and coordinate cluster response activities. Develop a community cluster response framework outlining the detection, response, and roles and responsibilities for responding to a suicide cluster. Establish a multidisciplinary team responsible for responding to a suicide cluster.
Routine monitoring of suicide, suicide attempts, and cluster detection	Identify suspected suicide clusters through routine monitoring of coronial data, emergency department data, and through local stakeholders. Using data to identify the characteristics of the suicide cluster and the psychosocial links between those who have died.
Coordination with the media and monitoring social media	Coordinate with local media for safe reporting of suicide. Monitor media and social media for potentially harmful content (e.g., information about the method or location of suicide). Monitor social media for individuals who may be at risk of suicide.
Identifying and supporting individuals at risk	Screen for individuals that may be at risk of suicide and suicide attempt. Refer individuals at risk to relevant services in the community. Provide support to those immediately bereaved by the suicides as well as those who are affected by the suicide deaths.
Promoting help-seeking and building community resilience	Promote help-seeking across the community, including at public memorial sites. Upskill the community in emotional health, wellness, and suicide prevention. Provide debriefing opportunities to stakeholders involved in the response to the suicide cluster.
Long-term follow-up and evaluation	Provide follow-up to those bereaved at anniversaries and other significant events related to the deceased. Identify what did or did not work in response to the suicide cluster.

**Table 2:** Results of the stakeholder survey based on the core components of cluster response frameworks.

Cluster Response Component	Response (N = 26) n (%)
**Preparing for a suicide cluster**	
The community had a pre-existing suicide cluster response plan at the time the suicide cluster emerged	24 (92%)
A lead/host agency was assigned to facilitate a coordinated response to the suicide cluster	17 (69%)
**Routine monitoring and cluster detection**	
Information received through existing data surveillance system	9 (35%)
Information received through word of mouth	17 (69%)
**Coordination with the media and monitoring social media**	
A media strategy for the safe reporting of suicide was implemented	21 (80%)
There was a specific person/official role responsible for overseeing media activities	11 (42%)
Social media activities were monitored during the suicide cluster	15 (58%)
Training and resources were provided to media professionals about the safe reporting of suicide	12 (47%)
**Identifying and supporting individuals at risk**	
Relatives and close friends provided bereavement support	24 (92%)
Procedures were implemented to identify high-risk individuals	18 (69%)
Referral pathways were identified and promoted	22 (83%)
There was a system in place to monitor high-risk individuals’ engagement with services	6 (23%)
**Promoting help-seeking and building community resilience**	
Help-seeking was promoted at shrines/public memorials	15 (58%)
Screening for suicide risk was conducted at public memorials and related events	8 (31%)
Gatekeeper training was provided to the community	21 (80%)
Community gatekeepers were notified to identify individuals at risk of suicide	20 (77%)
Stakeholders who responded to the suicide cluster had an opportunity to debrief and take care their own wellbeing	11 (42%)
**Long-term follow-up and evaluation**	
Procedures were implemented to follow up with bereaved individuals at important timepoints (e.g., birthdays and anniversaries)	17 (69%)
Response to the suicide cluster was evaluated.	12 (47%)

## Data Availability

The dataset used and analysed during the current study available from the corresponding author on reasonable request.

## Supplementary Materials

The following supporting information can be downloaded at: https://www.mdpi.com/article/10.3390/ijerph19084444/s1, Table S1: The core components and overlapping themes identified in existing cluster response frameworks.
