# Supplementary material for: Responding to Suicide Clusters in the Community: What Do Existing Suicide Cluster Response Frameworks Recommend and How Are They Implemented?
Source: Int J Environ Res Public Health. 2022 Apr 7;19(8):4444. doi: 10.3390/ijerph19084444 (PMC9031396; doi:10.3390/ijerph19084444)
Supplement: Supplementary file 1 [file ijerph-19-04444-s001.zip › ijerph-1612705-supplementary.pdf]

Table S1: The core components and overlapping themes identified in existing cluster response frameworks.

	CASA, New Zealand	Centre for Health Policy Programs and Economics, Australia	Public Health England, UK	Centers for Disease Control and Prevention, USA	Health Services Ireland
**1. Preparing for a suicide cluster**					
Assign a lead agency to oversee and coordinate cluster response activities.	*	*	*	*	
Develop a community cluster response framework outlining the detection, response, and roles and responsibilities for responding to a suicide cluster.	**	**	**	*	*
Establish a multidisciplinary team responsible for responding to a suicide cluster.	***	***	***	*	*
Establish communication agreements between stakeholders and agencies in the community for the detection and response to a suicide cluster.	**	*	*	*	*
Enlist members from local cultural groups in the community to be involved in preparing for a cluster.	***	*			*
Assign a lead agency to oversee and coordinate cluster response activities.	*	*	*	*	*
**2. Routine monitoring of suicide, suicide attempts and cluster detection**					
Initiate an investigation into a potential suicide cluster using official data sources such as coronial data, emergency department data, and information from agencies and local stakeholders.	**	*	*		
Investigate the characteristics of the suicides, suicide attempts, or self-harm, including method, and psychosocial links between those who have died.	***		**		
**3. Coordination with the media and monitoring social media**					
Coordinate with local media for safe reporting of suicide.	***	***	***	*	***
Assign a coordinator or spokesperson responsible for corresponding with the media.	*	*	*	*	*
Enlist local media to publish sources of help and promote recovery in the community.	***				**
Monitoring media and social media for potentially harmful content.	**	*	*		

Utilise social media to promote help-seeking resources.	*	*	***		*
Monitor social media for individuals who may be at risk of suicide.	*	*	**		
Dispel rumours and misinformation about the suicide deaths.	*	*			
**4. Identifying and supporting individuals at risk**					
Identify family and close friends immediately bereaved and provide screening and referral services for support.	**	*	***	*	*
Enlist members of the community to help identify individuals who may be at risk of suicidal behaviour.	**	*		*	
Identify and screen persons who may be high-risk of suicide.	**	*	***		*
Provide referral to counselling services for those affected.	*	*	*	*	*
Have a plan to scale up access to counselling services.	**	*			*
Establish community support networks for those affected (e.g., via GPs or other local agencies)	**	*	**		*
**5. Promoting help seeking and building community resilience**					
Adapt the environment to include means restriction if applicable.	*	*	***	*	
Establish an ongoing surveillance system for suicide and suicide attempt.	*	*	*		
Develop a strategy for improving services or other community level risk factors that contributed to the suicide cluster.	*	**			*
Provide skills and training opportunities in emotional health, wellbeing, and suicide prevention to the public and to service providers and individuals who responded to the suicide cluster.	***	*			
**6. Long term follow-up and evaluation**					
Plan for 12-month anniversaries and other significant events related to the deceased.	***	*	*		
Provide Follow-up screening to high-risk individuals.	*		*		
Provide Debriefing to stakeholders who responded to the suicide cluster.	**	*	*		
Review the cluster response activities and update the plan based on what worked or what didn’t work	*	*	*		*
Consult with stakeholders and community members about their experience with the cluster response.	*	*	*		*

Debrief stakeholders who responded to the suicide cluster.	*	*	*		*

* The core component is included in the cluster response framework
** The core component is demonstrated in a case study or example scenario to assist with implementation
*** Implementation of the core component is facilitated through the inclusion of templates, worksheets or other practical resource
